# Ultrasound Assisted Extraction of Phenolic Compounds from Peaches and Pumpkins

**DOI:** 10.1371/journal.pone.0148758

**Published:** 2016-02-17

**Authors:** Ammar Altemimi, Dennis G. Watson, Ruplal Choudhary, Mallika R. Dasari, David A. Lightfoot

**Affiliations:** 1 Department of Plant, Soil and Agricultural Systems, Southern Illinois University at Carbondale, Carbondale, IL 62901, United States of America; 2 Department of Food Science and Biotechnology, College of Agriculture, University of Basrah, Basrah, Basrah Province, Iraq; 3 Department of Chemistry and Biochemistry, Southern Illinois University at Carbondale, Carbondale, IL 62901, United States of America; Agroecological Institute, CHINA

## Abstract

The ultrasound-assisted extraction (UAE) method was used to optimize the extraction of phenolic compounds from pumpkins and peaches. The response surface methodology (RSM) was used to study the effects of three independent variables each with three treatments. They included extraction temperatures (30, 40 and 50°C), ultrasonic power levels (30, 50 and 70%) and extraction times (10, 20 and 30 min). The optimal conditions for extractions of total phenolics from pumpkins were inferred to be a temperature of 41.45°C, a power of 44.60% and a time of 25.67 min. However, an extraction temperature of 40.99°C, power of 56.01% and time of 25.71 min was optimal for recovery of free radical scavenging activity (measured by 1, 1-diphenyl-2-picrylhydrazyl (DPPH) reduction). The optimal conditions for peach extracts were an extraction temperature of 41.53°C, power of 43.99% and time of 27.86 min for total phenolics. However, an extraction temperature of 41.60°C, power of 44.88% and time of 27.49 min was optimal for free radical scavenging activity (judged by from DPPH reduction). Further, the UAE processes were significantly better than solvent extractions without ultrasound. By electron microscopy it was concluded that ultrasonic processing caused damage in cells for all treated samples (pumpkin, peach). However, the FTIR spectra did not show any significant changes in chemical structures caused by either ultrasonic processing or solvent extraction.

## Introduction

The definition of an antioxidant is a bioactive compound which can inhibit or delay the oxidation of other molecules. Antioxidants are categorized into natural and synthetic antioxidants [1–2]. Commonly used synthetic antioxidants include butylated hydroxytoluene (BHT), butylated hydroxyanisole (BHA), propylgallate and tertbutylhydroquinine. Reports of negative effects of synthetic chemical preservatives on human health has led to a desire to replace these synthetic chemical preservatives with natural preservatives showing antioxidant and/or antimicrobial activities [3–5]. The use of nontoxic natural preservatives has increased in parallel with increased consumer awareness about these preservatives [6].

Peach (*Prunus persica* L.) is an economical fruit in many countries. Many studies have shown the phenolic compounds of diverse peach genotypes are major sources of antioxidants [7]. Both raw and canned peaches inhibited low-density lipoprotein (LDL) oxidation. Antioxidant activity protected 56–87% of the LDL. Protection was attributed to the hydroxycinnamic, chlorogenic and neochlorogenic acids in peaches [8–10].

Pumpkins belong to the *Cucurbitaceae* family that are classified by species depending on the texture and shape of their stems to the *Cucurbita pepo*, *C*. *moschata*, *C*. *maxima* and *C*. *mixta* [11]. Numerous studies confirmed that pumpkin consumption can regulate metabolism inside the human body and reduce toxins [12]. Pumpkins have become a part of healthy diet partly because of their high carotenoid contents [13]. Recently, new techniques such as supercritical fluid extraction (SFE), microwave assisted extraction (MAE), and ultrasound-assisted extraction (UAE) have been used for the extraction of phenolic compounds from plants. Among all of these techniques [14–15], UAE was widely employed to extract bioactive compounds from plant materials due to the high extraction efficiencies that can be achieved at relatively low temperatures [16]. UAE is inexpensive so it is a good alternative to conventional extraction techniques. Ultrasound waves helped disrupt plant cell walls, improved the solvent penetration and enhanced mass transfer across cell membrane [17]. The result was higher extract yields. Advantages of UAE in food processing include extending the shelf life of products, consuming less energy, decreasing the processing time for extracts increasing the bioactivity of the phenolics and enhancing food quality [18].

Response surface methodology (RSM) has been applied to optimize ultrasonic parameters (i.e. extraction temperature, power percentage and exposure time) of phenolic compound extraction in prior studies [17–20]. While UAE may be expected to improve the extraction yield of phenolic compounds of fruits like peach and pumpkin, research was needed to provide recommendations on optimum conditions, such as frequency, time, temperature, and power level for UAE extractions.

The objectives of this study were to investigate; (1) the effect of UAE frequency on yield of phenolics and antioxidant activity; (2) the response surface models; (3) the effect of extraction parameters on total phenolic content; (4) the effect of extraction parameters on antioxidant activity; (5) the use of predictive models; (6) the chemical structures of samples before and after processing, by Fourier transformed infrared spectroscopy; and (7) the effects of UAE on cell structure by scanning electron microscopy.

## Materials and Methods

### Sample preparation

Fresh peaches (‘*Red Haven’*) and pumpkins (‘*Libbys Select’*) were harvested at maturity from several plants selected at random within a field at the Horticulture Research Center farm (37.712706; -89.261778) on Rowden Road near Southern Illinois University (Carbondale, IL). Peaches were obtained on July 25^th^, 2013 and pumpkins were obtained on September 20^th^, 2013. Peaches and pumpkins were grown according to conventional methods for southern Illinois. Synthetic nutrients and pesticides were applied according to recommendations for peach and pumpkin production in southern Illinois. The samples were provided by Dr. Alan Walters, of the Department of Plant, Soil and Agricultural Systems, College of Agricultural Sciences, Southern Illinois University, USA. The plants were cleaned and sliced into small pieces, and then sealed and stored in plastic bags in a refrigerator freezer (- 18 C) for five days before freeze-drying.

### Effect of ultrasound frequency on phenolic extractions

In a preliminary study, the efficiencies of ultrasonic frequencies of 37 and 80 KHz on the total phenolic and free radical scavenging activity of pumpkins and peaches were investigated. UAE was performed at 40°C (temperature), 50% (power) and 30 min (time). The optimal ultrasound frequency resulted in the highest total phenolic and was employed to optimize phenolic compound extraction from peaches and pumpkins.

### Solvent-extraction

The solvent extraction technique was used with slight adjustments [21]. Briefly, ten grams of lyophilized plants (peaches and pumpkins) were weighed and 100 mL of methanol was added in a 200ml glass flask. The mixture was placed in a water bath (Elmasonic P30, Elma Hans Schmidbauer GmbH, Sinden, Germany) for 30 min at 50°C to solubilize bioactive compounds from the plant materials.

### Ultrasonic extraction

An Elmasonic P30 (P30) ultrasonic cleaner was coupled with controlled heating using a cooling coil (Fisher Scientific Inc. St Louis USA); connected with a cooling chiller system; and a water pump (model HJ-111, submersible pump, flow rate 250 L/h, Sunsun Inc., Zhejiang, China). Coupled heating and cooling helped to maintain temperatures that were evenly distributed across the ultrasonic water bath. Extracts were made at 37 kHz frequency with three heated bath temperatures, and three power settings expressed as a percentage of full power (30–100%). The prior work of Altemimi et al. [22–24] with the same ultrasonic equipment was used as a guide and selected variables were bath temperatures of 30°C, 40°C, and 50°C; power level settings of 30%, 50%, and 70%; and ultrasonic duration of 10 min, 20 min, and 30 min. The manufacturer rated the P30 with an ultrasonic power rating of 120 W. The P30 had a proprietary algorithm to adjust power based on the impedance of the system. For a specific power setting, samples experienced the same degree of cavitation regardless of the load in the tank. For all treatments, the bath of the P30 contained 1.7 L of water before the treatment containers were added. Ultrasonic power was expressed as W/cm^2^, based on the power setting as a percentage of rated power and the volume of the bath solution prior to addition of treatment containers. Ultrasonic power for the 30%, 50%, and 70% power settings inside the extract containers were 21 W/cm^2^, 35 W/cm^2^, 49 W/cm^2^, respectively. These power settings were independently verified using a calorimetric method [25].

### Total phenolic compounds

The Folin–Ciocalteu assay was used to measure total phenolic compound according to [26]. Briefly, sodium carbonate was prepared by weighing and dissolving 2 gm into 100 ml distilled water. Exactly, 1 gm of the crude extracts was dissolved in 46 ml of distilled water with 1 ml of Folin-Ciocalteu solution. The mixture was shaken by using a Maxi mix (Barnstead Thermolyne, USA) for 10 min, and 3 ml of sodium carbonate (2% w/v) was added. The mixture was kept in the dark for two hours with intermittent shaking to be sure the whole mixture homogenized. The absorbance was measured at 750 nm, compared to a calibration curves prepared with known amounts of gallic acid (Roth, Karlsruhe, Germany). The results were expressed as mg/ 100 g dry gallic acid equivalent. Raw data is provided as Tables A-F in S1 File.

### Free radical scavenging activity

About 3 ml of prepared solution (dissolving 2 mg of 1, 1-diphenyl-2-picrylhydrazyl (DPPH) in 100 ml of methanol) was mixed with 1 ml of samples extracts according to Braca et al. [27]. All of the mixtures were kept for 30 min in the dark place, thereby the absorbance for each samples was measured by spectrophotometer at 517 nm. Methanol (1 ml) with 3 ml DPPH solution (0.002% w/v in methanol) was used as the blank. The optical density was determined and % inhibition was calculated using the formula given below:

$$\%\;\text{inhibition of DPPH activity}=\left[{\text{A}}_{\text{b}}{–\text{A}}_{\text{s}}{/\text{A}}_{\text{b}}\right]\;*\;100$$

Where as

A_b_: absorbance of control

A_s_: absorbance of sample

### Experimental designs

The effects of three independent variables on total phenolics and antioxidant activity (DPPH) were investigated by using response surface methodology (RSM). The main factors which can enhance extraction efficiencies were temperature °C (X_1_), power % (X_2_) and time min (X_3_). These independent variables were included to optimize the extraction process. In this study, the coded values of the experimental factors and their settings for the experimental design are shown in the Table 1; and the experimental data were as presented in the Table 2.

**Table 1:** Settings of variables for the experimental design.

Symbols	Independent variables	-1	0	1
X_1_	Temperature (C)	30	40	50
X_2_	Power (%)	30	50	70
X_3_	Time (min)	10	20	30

**Table 2:** Encoded design arrangement of the responses of total phenolic (TP) mg/ 100 g gallic acid equivalent and antioxidant activity (DPPH %) for pumpkin and peach extracts.

Run	TemperatureC (x1)	Power%(x2)	Timemin(x3)	pumpkin		Peach	
				TP*	DPPH*	TP*	DPPH*
26	30	30	10	38.84±0.402	57.67±0.487	50.22±0.203	69.19±0.188
14	30	30	20	39.91±0.526	60.18±0.035	50.23±1.021	69.93±0.514
19	30	30	30	39.9±0.970	58.93±1.026	50.78±0.955	69.44±0.777
27	30	50	10	38.46±0.669	57.90±0.076	50.07±0.870	69.14±1.231
17	30	50	20	40.94±0.050	60.31±0.577	51.73±0.063	70.95±0.060
8	30	50	30	40.26±0.045	60.16±0.045	51.47±0.307	70.11±0.819
7	30	70	10	39.51±0.618	60.14±0.050	50.63±0.647	69.11±0.121
15	30	70	20	40.81±0.548	60.48±0.566	51.88±0.538	70.48±0.476
24	30	70	30	40.25±0820	60.84±0.448	51.35±0.840	70.29±0.810
21	40	30	10	42.31±0.181	62.25±0.227	53.37±0.140	72.22±0.262
1	40	30	20	41.77±1.442	61.76±1.516	52.76±1.431	71.43±1.046
12	40	30	30	42.69±0.277	62.69±0.277	53.62±0.225	72.69±0.277
6	40	50	10	41.55±1.500	62.49±2.268	52.70±1.413	71.55±1.500
18	40	50	20	43.85±1.706	64.85±0.046	54.41±1.485	73.81±1.940
23	40	50	30	44.09±1.090	64.43±2.046	54.82±0.581	73.79±1.219
16	40	70	10	42.32±0.092	62.24±0.050	53.52±0.202	71.96±0.592
3	40	70	20	42.71±0.817	64.01±0.401	53.34±1.137	71.71±0.572
5	40	70	30	42.08±1.134	63.07±0.113	52.99±1.038	71.76±0.631
11	50	30	10	40.05±0.195	62.03±3.308	50.97±0.374	70.03±0.232
2	50	30	20	41.64±0.869	61.64±0.869	52.69±0.918	71.31±1.271
13	50	30	30	42.19±0.189	62.22±0.049	53.23±0.326	72.20±0.183
20	50	50	10	40.73±0.430	60.09±0.120	51.77±0.426	70.73±0.430
9	50	50	20	41.44±0.472	61.44±0.477	52.32±0.332	71.41±0.409
22	50	50	30	42.01±0.808	61.94±0.140	52.27±1.859	72.02±0.814
10	50	70	10	39.06±0.238	59±1.095	50.31±0.420	69.39±0.463
4	50	70	20	40.21±0.092	60.55±0.610	51.26±0.125	70.22±0.098
25	50	70	30	39.99±0.671	61.58±1.031	51.22±0.620	70.01±0.671

*Mean ± standard deviation (n = 3)

The complete design was carried out in random order and consisted of 27 combinations including three replicates (Table 2). The data from the experimental design were analyzed by multiple regressions to fit the following quadratic polynomial model:

$$Y=b_{0}\sum_{i=1}^{3}b_{i}X_{i}+\sum_{i=1}^{3}b_{ii}{X^{2}}_{i}+\sum_{i\neq j=1}^{3}b_{ii}X_{i}X_{j}$$ (1)

Where Y is the predicted response; b_0_ is the intercept; b_1_, b_2_ and b_3_ are the linear coefficients of temperature (X_1_), power (X_2_) and time (X_3_), respectively; b_11_, b_22_ and b_33_ are the squared coefficient of temperature of sonication, power and time respectively; b_12_, b_13_ and b_23_ are the interaction coefficients of temperature of sonication, power and time respectively. Then, the settings of the independent variables were represented as X_i_ and X_j_.

### Tests of the validity of the models

The independent variables, extraction temperature, ultrasonic power, and extraction time were optimized by using the response surface methodology (RSM) thereby the total phenolic and the rate of DPPH radical scavenging were measured using pumpkin and peach extracts under the optimum ultrasonic conditions. Comparisons among the predicted values and the experimental results were made in order to test the models developed.

### Scanning electron microscopy (SEM)

The SEM was conducted to study the morphological analysis of pumpkin and peach samples before and after processing. The analysis was carried out with a scanning electron microscope (SEM, Quanta 450 FEG, FEI Inc., Hillsboro, Oregon, USA). Samples were sputter-coated with a thin layer of gold-palladium (6–11 nm; 10 mA; 40 s) at room temperature before imaging.

### Fourier-transformed infrared (FTIR) spectra.

KBr powder was mixed with ultra-sonicated pumpkin, heated pumpkin, and non-processed pumpkin separately in order to prepare a slurry at 1% (w/v) concentrations. A KBr disc was prepared for FTIR by pressuring to approximately 5.5 tons for 3 min. This method was replicated twice for peach samples. At a resolution of 5 cm^−1^spectra were obtained (Nicolet 6700, Thermo Scientific, St Louis, USA) and recorded over the mid infrared range of 500–4,000– cm^−1^.

### Statistical analysis

Design-Expert^TM^ software (version 9) was used to analyze the experimental results with the response surface design (State-Ease Inc. Minneapolis, MN, USA). Using p-values less than 0.05 was considered statistically significant. One way ANOVA was assigned to test if there is any difference between the experimental and predicted values.

## Results and Discussion

### Effect of ultrasonic frequency on the total phenolic content and the rate of DPPH free radical scavenging

**Fig 1:**
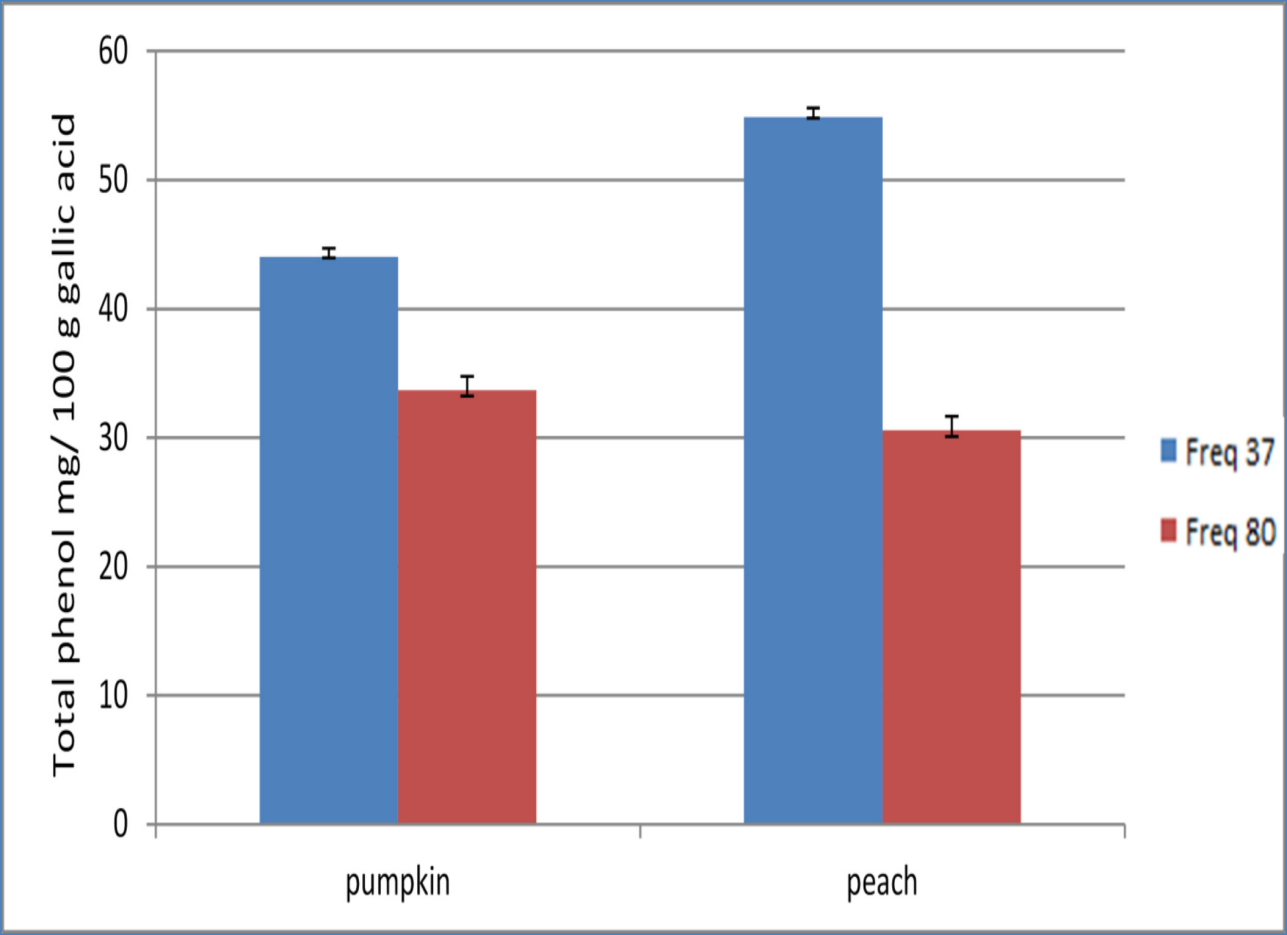
The effect of ultrasound frequency on total phenolic content.

**Fig 2:**
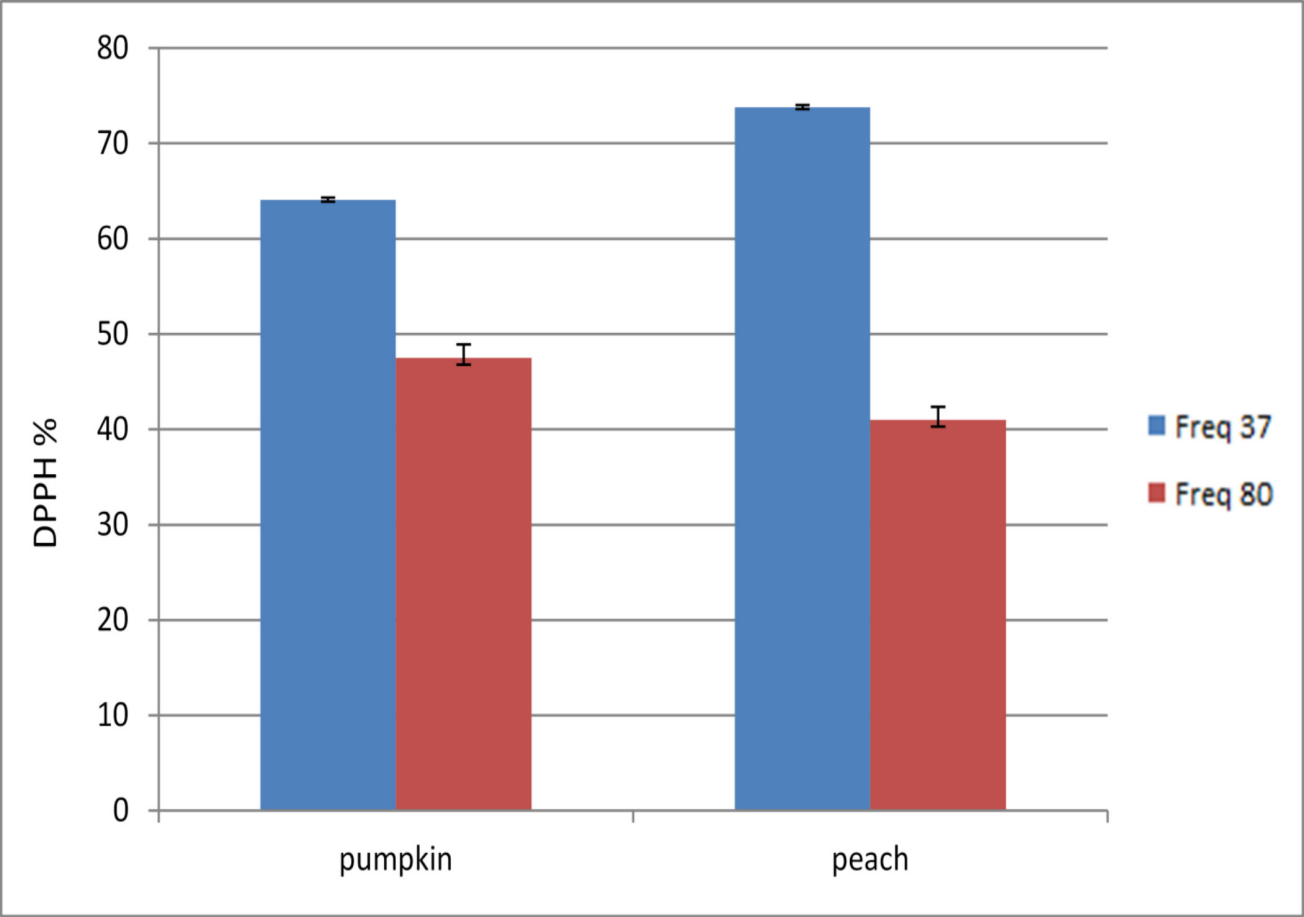
The effect of ultrasound frequency on % DPPH.

Ultrasound frequency was an important factor in extracting bio active compounds from plant material (Fig 1). The statistical analyses showed that there was a significant difference (p< 0.05) between 37 kHz and 80 kHz. The results showed that the highest total phenolic and DPPH free-radical scavenging rate in both pumpkin and peach extracts was at 37 kHz (Fig 2). This suggested that the higher frequency (80 kHz) may cause the collapse of bubbles in the samples. Consequently, high frequencies did not allow sufficient time for cavitation bubbles to extract all the target compounds. The results were in agreement with Liu et al.[28] who found the 45 kHz was superior compared to 80 and 100 kHz. Subsequent treatments (see Table 2) were completed at 37 kHz.

### The models of response surfaces

Table 2 shows the total phenolic (TP) mg/ 100 g gallic acid equivalent and antioxidant activity (DPPH % reduction) for pumpkin and peach extracts from all experiments. The quadratic polynomial model was assigned based on the results in Table 2 in order to perform multiple regression analysis. The analysis of variance (ANOVA) and regression coefficients are shown in Table 3 and indicate the contribution of the quadratic model [19]. The lack-of-fit (p > 0.05) was used to test the contribution of the quadratic model “fitness”. The values indicated the suitability of models to accurately predict the variation.

**Table 3:** Results of ANOVA and the regression coefficients.

Source	Pumpkin		Peach	
	TP	DPPH	TP	DPPH
*b*_0_	43.37	63.72	54.05	73.09
X_1_	0.47	0.75	0.43	0.48
X_2_	-0.13	0.16	-0.076	-0.20
X_3_	0.59	0.67	0.45	0.50
$X_{1}^{2}$	-2.25	-2.67	-2.15	-1.99
$X_{2}^{2}$	-0.58	-0.31	-0.49	-0.76
$X_{3}^{2}$	0.57	-0.64	-0.33	-0.38
X_1_X_2_	-0.54	-0.82	-0.56	-0.43
X_1_X_3_	0.063	0.034	0.083	0.14
X_2_X_3_	-0.18	0.19	-0.16	-0.11
R^2^	0.744	0.713	0.704	0.70
P-value	0.001	0.0012	0.001	0.0001
F-value	22.94	19.66	18.77	18.45
Lack of fit	0.0518	0.0747	0.2970	0.1025
F-value for lack-of-fit	1.80	1.69	1.20	1.58

### Effect of ultrasonic parameters on total phenolic content of pumpkin

The fitted quadratic surface models for total phenolics in pumpkin extracts by ANOVA and regression coefficients are shown in Table 3. The quadratic regression model of total phenolic showed that the coefficient of determination (R^2^) was 0.744 while the value of the adjusted coefficient of determination coefficient (R^2^ adj) was 0.711, resulting in a high degree of correlation between the observed and predicted values. A low coefficient of variation (CV) of 2.05% suggested good precision and high reliability of the models to predict experimental results. The F-value of 22.94 implied the model was significant. There was only a 0.1% chance that an F-value this large could occur due to chance or noise. The “lack-of-fit F-value” of 1.80 implied that the lack-of-fit was not significant relative to the pure error. There was a 5.18% chance that a “lack-of-fit F-value” this large could occur due to chance or noise, which indicated that the model equation was adequate for predicting the total phenolics. The P-value less than 0.05 indicated that the model terms were significant.

**Fig 3:**
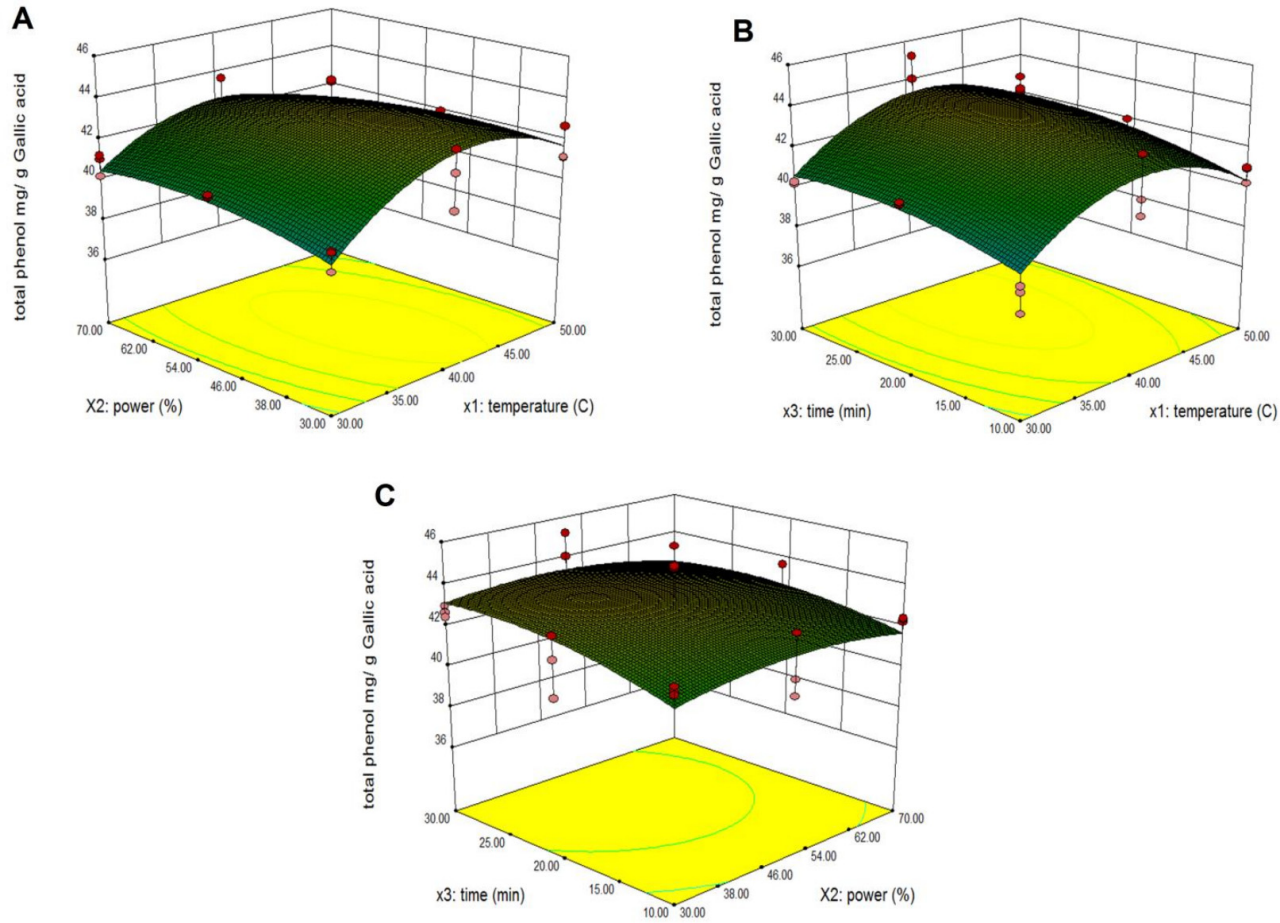
Response surface and contour plots for the effect of independent variables on total phenolics from pumpkin extracts. Panel (A) power and temperature. Panel (B) time and temperature. Panel (C) time and power.

Response surface models sufficiently predicted the effects of parameter variables (ultrasonic temperature, power and extraction time) and their interactions on total phenolics of pumpkin extracts. The third variable was assigned to be constant at the intermediate setting while surface plots of three-dimensions were shown by two independent variables. As shown in Fig 3A, when the extraction time (X_3_) was fixed at its intermediate point (20 min), it was predicted that maximum total phenolic extraction could be achieved when the combination of extraction temperature and power were 41.45°C and 44.60% respectively. The total phenolics increased with an increase in extraction temperature from 30°C to 41.45°C. However, the total phenolic content decreased when the extraction temperature was above 41.45°C. This might be ascribed to the capability of lower temperatures to release phenolic compounds in the mixture more effectively. The total phenolic yields increased with increasing extraction power from 30% to 44.66% within 41.45°C of temperature extraction. The above results agreed with Ma et al. [29] who confirmed the positive effects of increasing the setting of power on the yields of phenolic compounds from citrus peel. The interaction of extraction temperature and time are presented in Fig 3B. The increased extraction of total phenolics was observed with an increase of ultrasonic time from 10 min to 25.67 min, probably because an extended extraction time favors the extraction of phenolic compounds [30]. The interaction of extraction power and extraction time are presented in Fig 3C It was found that maximum total phenolics were achieved when the extraction time was 25.67 min and the extraction power was 44.60%. This finding was in agreement with Qu et al. [20] who discovered that the extraction yield of phenolics increased when time increased from 5 min to 15 min within optimization of ultrasonic extractions of polysaccharides from *Ziziphus jujuba (*Mill) by response surface methodology. In contrast, conventional methods such as ethanol and boiling water extraction may require up to 2 h to reach peak efficiencies thus increasing the extract efficiency. Ultrasound water baths produce sufficient cavitation to create shear forces to break the cell walls. Further, sonication increases the diffusion of cell contents into the extraction solution. Jerman et al.[31] showed the ultrasonication method of extraction can enhance the yield of phenolic compounds from olive by up to 80% in a short time compared to conventional solvent extraction.

### Effect of ultrasonic parameters on DPPH of pumpkin

The fitted quadratic surface models for DPPH % of pumpkin extracts by ANOVA and regression coefficients are shown in Table 3. The quadratic regression model of DPPH showed that the coefficient of determination (R^2^) was 0.713 while the value of the adjusted coefficient of determination coefficient (R^2^ adj) was 0.677 showing a high degree of correlation between the observed and predicted values. The CV of 1.81% suggested a good precision and higher reliability of the models to predict experimental results. The F-value of 19.66 implied the model was significant. There is only 0.12% chance that an F-value this large could occur. The “lack-of-fit F-value” of 1.69 implied that the lack-of-fit was not significant relative to the pure error. There was a 7.47% chance that a “lack-of-fit F-value” this large could occur, which indicated that the model equation was adequate for predicting the rate of DPPH free radical scavenging. The P-value less than 0.05 indicated that the model was significant.

**Fig 4:**
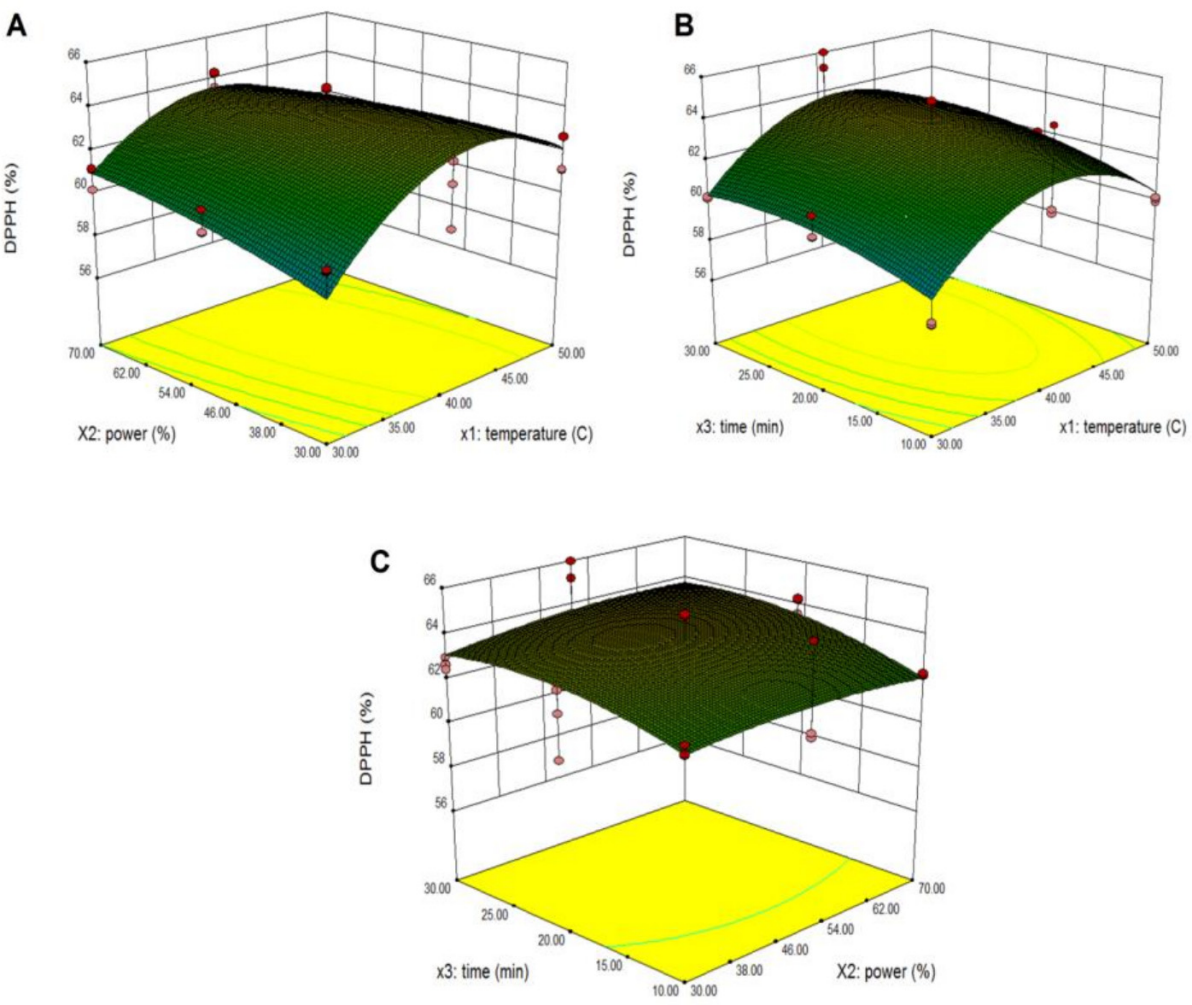
Response surface and contour plots for the effect of independent variables on % of DPPH from pumpkin extracts. Panel (A) power and temperature. Panel (B) time and temperature. Panel (C) time and power.

Response surface models were used to study the effects of parameter variables (ultrasonic temperature, power and extraction time) and their interactions on % DPPH of pumpkin extracts. The third variable was assigned to be constant at the intermediate point while surface plots of three-dimensions were made by two independent variables. As shown in Fig 4A, when the extraction time (X_3_) was fixed at its intermediate point (20 min), maximum predicted % DPPH was at the extraction temperature and power of 40.99 C and 56.01%. The % DPPH increased with an increase in extraction temperature from 30°C to 40.99°C. However, the % DPPH decreased when the extraction temperature was above 46.34°C probably because the temperature led to the loss of some labile compounds with high antioxidant capacity. The % DPPH increased with increasing extraction power from 30% to 56.01%. The interaction of extraction temperature and time are presented in Fig 4B. Increasing % DPPH was observed during an increase of ultrasonic time from 10 min to 25.71 min at 40.99°C. The interaction of extraction power and extraction time are presented in Fig 4C. It was found that the maximum rate of % DPPH was achieved when the extraction time was 25.71 min and the extraction power was 56.01%. This findings were in agreement with Hossain et al. [17] who showed that increasing antioxidant activity was related to having appropriate power and time settings to disrupt plant cell walls, thus enhancing mass transfer across the cell membrane. Moreover, Ghasemzadeh et al. [32] found that the DPPH radical scavenging activity of the extracts were ranked in the following order: ethanol-water (50:50 v/v) ultrasonic (84.21%), ethanol-water (50:50 v/v) maceration (71.41%), ethanol ultrasonic (68.05%), and ethanol maceration (57.33%) methods. Their results showed that the DPPH radical scavenging activity of Hashemi rice bran was enhanced and improved by the ultrasonic extraction method compared to a different extraction technique (reflux) and solvent (methanol) for extraction. The ultrasonic energy penetrates and extracts most of the available bioactive compounds thereby reducing the losses that may happen using other methods.

### Effect of ultrasonic parameters on total phenolic content of peach

The fitted quadratic surface models for total phenolics in peach extracts by ANOVA and regression coefficients are shown in Table 3. The quadratic regression model of total phenolics showed that the coefficient of determination (R^2^) was 0.704 while the value of the adjusted coefficient of determination coefficient (R^2^ adj) was 0.666. Therefore, there was a high degree of correlation between the observed and predicted values. The CV of 1.64% suggested a good precision and higher reliability of the models to predict experimental results. The F-value of 18.77 implied the model was significant. There was a 0.1% chance that an F-value this large could occur by chance. The “lack-of-fit F-value” of 1.20 implied that the lack-of-fit was not significant relative to the pure error. There was a 29.70% chance that a “lack-of-fit F-value” this large could occur, which indicated that the model equation was adequate for predicting the total phenolics. The P-value less than 0.05 indicated that the model was significant.

**Fig 5:**
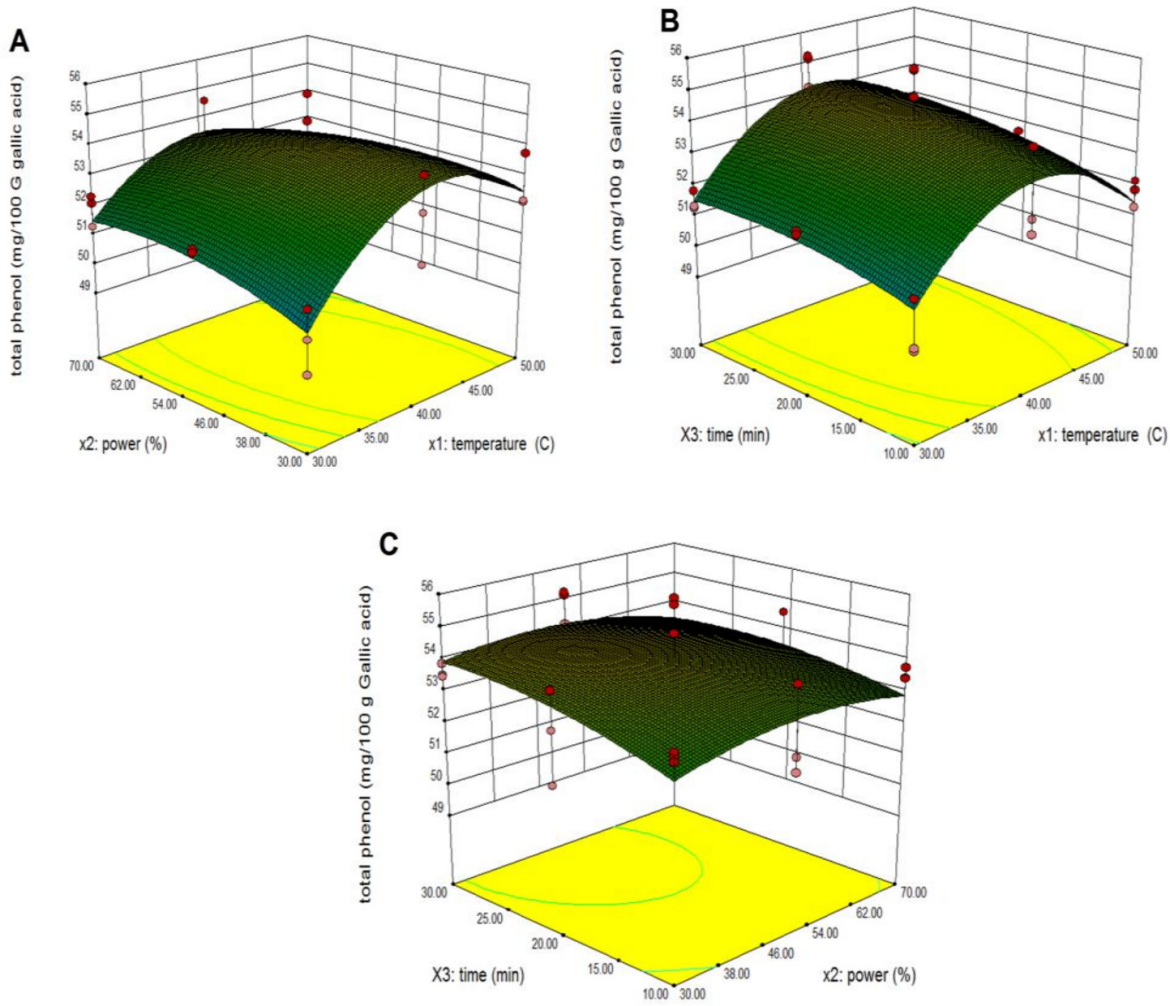
Response surface and contour plots for the effect of independent variables on total phenolics from peach extracts. Panel (A) power and temperature. Panel (B) time and temperature. Panel (C) time and power.

Response surface models were plotted to study the effects of parameter variables (ultrasonic temperature, power and extraction time) and their interactions on the total phenolic in peach extracts. The third variable was assigned to be constant at the intermediate point while surface plots of three-dimensional were showed by two independent variables. As shown in Fig 5A, when the extraction time (X_3_) was fixed at its intermediate point (20 min), that the predicted maximum total phenolics extraction would be achieved when extraction temperature and power were 41.53°C and 43.99%. The total phenolics increased with an increase in extraction temperature from 30°C to 41.53°C. In contrast, decreasing the total phenolic content was observed when the extraction temperature was above 41.53°C. This might be due to an increase in the ultrasonic temperatures which can increase diffusivity of the solvent into cells and enhance desorption and solubility of target compounds from the cells, thereby increasing the efficiency of extraction [31]. An increased total phenolic yield was obtained by increasing the extraction power from 30% to 43.99% at 41.53°C. This finding was in agreement with Toma et al. [32] who mentioned that the acoustic wave can principally cause a breakage in the biological cell and enhance the release of cell content into the extraction solvents. The interaction of extraction temperature and time are presented in Fig 5B. The increased extraction of total phenols was observed with an increase of ultrasonic time from 10 min to 27.89 min. The findings were in agreement with Muiz-Marquez et al. [33] who found that the phenolic compounds yield from lyophilized *Laurus nobilis* L. increased when extraction time was increased. The interaction of extraction power and extraction time is presented in Fig 5C. It was found that maximum total phenols were achieved when the extraction time was 27.89 min and the extraction power was 43.99%.

### Effect of ultrasonic parameters on rate of DPPH of peach

The fitted quadratic surface models for rate of DPPH of peach extracts by ANOVA and regression coefficients are shown in Table 3. The quadratic regression model of total phenolics showed that the coefficient of determination (R^2^) was 0.70 while the value of the adjusted coefficient of determination coefficient (R^2^ adj) was 0.662 showing a high degree of correlation between the observed and predicted values. The CV of 1.20% suggested good precision and high reliability of the models to predict experimental results. The F-value of 18.45 implied the model is significant. There was only 0.1% chance that an F-value this large could occur due to noise. The “lack-of-fit F-value” of 1.58 implied that the lack-of-fit was not significant relative to the pure error. There was a 10.26% chance that a “lack-of-fit F-value” this large could occur due to noise, which indicated that the model equation was adequate for predicting the rate of DPPH. The P-value less than 0.05 indicated that the model was significant

**Fig 6:**
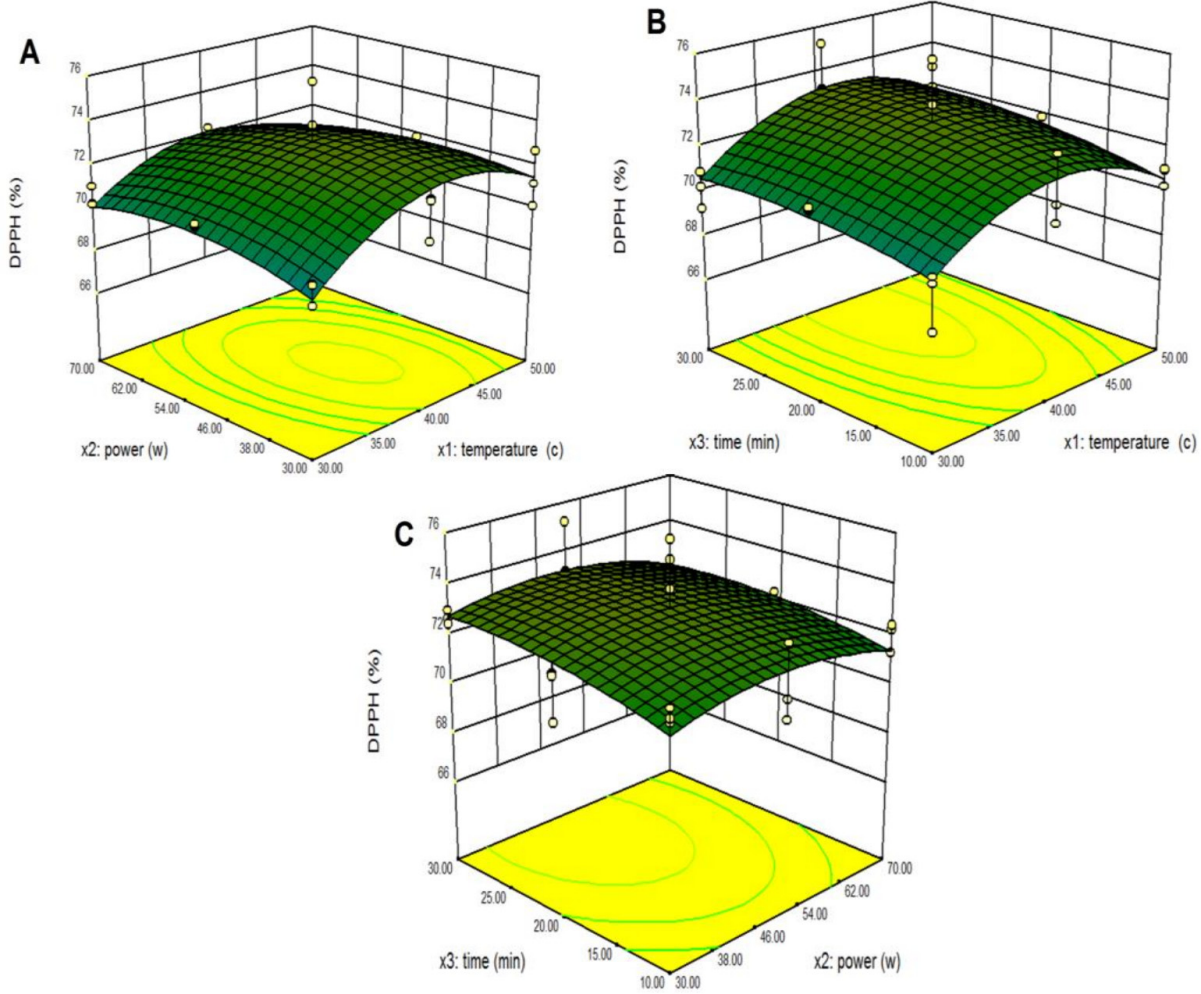
Response surface and contour plots for the effect of independent variables on % of DPPH from peach extracts. Panel (A) power and temperature. Panel (B) time and temperature. Panel (C) time and power.

In order to evaluate the effects of independents variables (ultrasonic temperature, power and extraction time) and their interactions on rate of DPPH of peach extracts, response surface models were assigned. The third variable was assigned to be constant at the intermediate setting while three-dimensional surface plots were shown by two independent variables. As shown in Fig 6A, when the extraction time (X_3_) was fixed at its intermediate setting (20 min), it can be concluded that maximum total phenols extraction could be achieved when the combination of extraction temperature and power were 41.60°C and 44.88% respectively. The rate of DPPH increased with an increase in extraction temperature from 30°C to 41.60°C. However, the rate of DPPH decreased when the extraction temperature was above 41.60°C. This might have happened because of the degradation of phenolic compounds when assigning high power and high temperature, thereby producing cavitation bubble collapse [34–36]. The interaction of extraction temperature and time are presented in Fig 6B. The increased rate of DPPH was observed with an increase of ultrasonic time from 10 min to 27.49 min within 41.60°C of extraction temperature. The interaction of extraction power and extraction time is presented in Fig 6C. It was found that maximum DPPH was achieved when the extraction time was 27.49 min and the extraction power was 44.88%. This result agreed with Qu et al. [20] who confirmed that the hydroxyl radical scavenging activity was high at a low ultrasonic power with an extraction time of 15 min. A comparison between ultrasonic extraction method and the solvent extraction methods was reported by Wu et al.[37]. The researchers tried to extract the aroma compounds from the root of ginseng by using ultrasonic treatment and solvent methods. Their results showed that the compounds obtained by ultrasound method were three fold more stable than the compounds produced by the solvent extraction methods. Thus, the percentage of DPPH was higher compared to solvent methods without ultrasounds.

### Optimization of ultrasonic parameters and verification of predictive models

**Fig 7:**
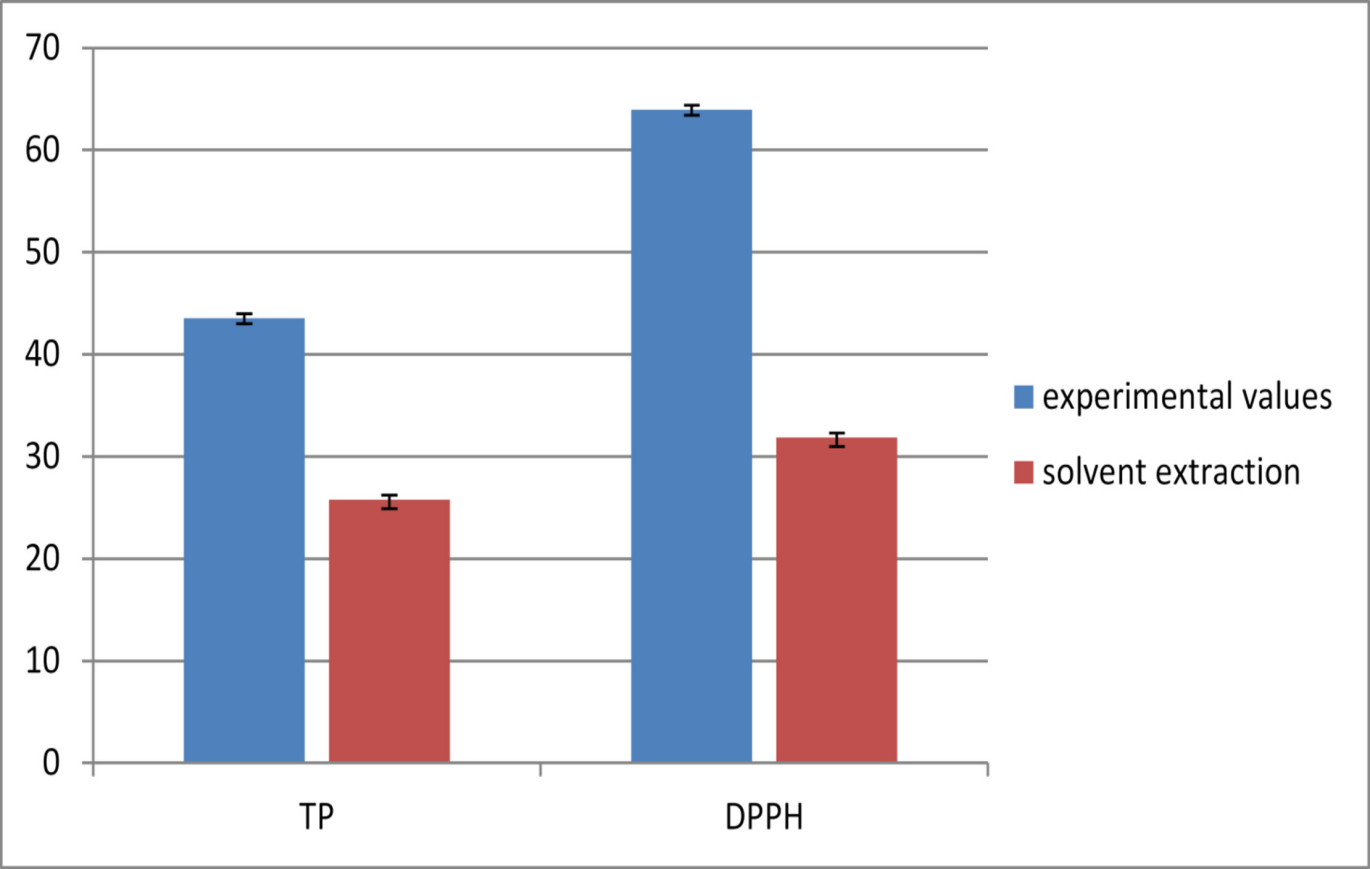
Experimental values and solvent extraction of TP(mg/100 g gallic acid) and DPPH (%) obtained for pumpkin extracts.

Performing an optimization design was related to the experimental results in order to evaluate the optimal extraction condition to measure the total phenolics and the rate of DPPH for pumpkin and peach extracts. The goal was to get the highest values of the total phenolic compounds and DPPH for pumpkins and peach extracts. Therefore, for pumpkins extracts, there were two optimal extraction conditions which were established to get the highest values: (1) For total phenolics; modify the extraction temperature of 41.45°C to 42°C, and extraction power from 44.60% to 45% and extraction time from 25.67 min to 26 min. (2) For rate of DPPH: modify the extraction temperature of 40.99C to 41°C, and extraction power from 56.01% to 56%; and extraction time from 25.71min to 26 min. The results are shown in Fig 7 with the amounts of total phenolics and % DPPH respectively under the optimal conditions and solvent extraction conditions. There was no significant difference (p > 0.05) between the experimental and predicted values of total phenolics and the rate of DPPH. Hence, the models can be used to optimize the process of phenolic extraction from pumpkins.

**Fig 8:**
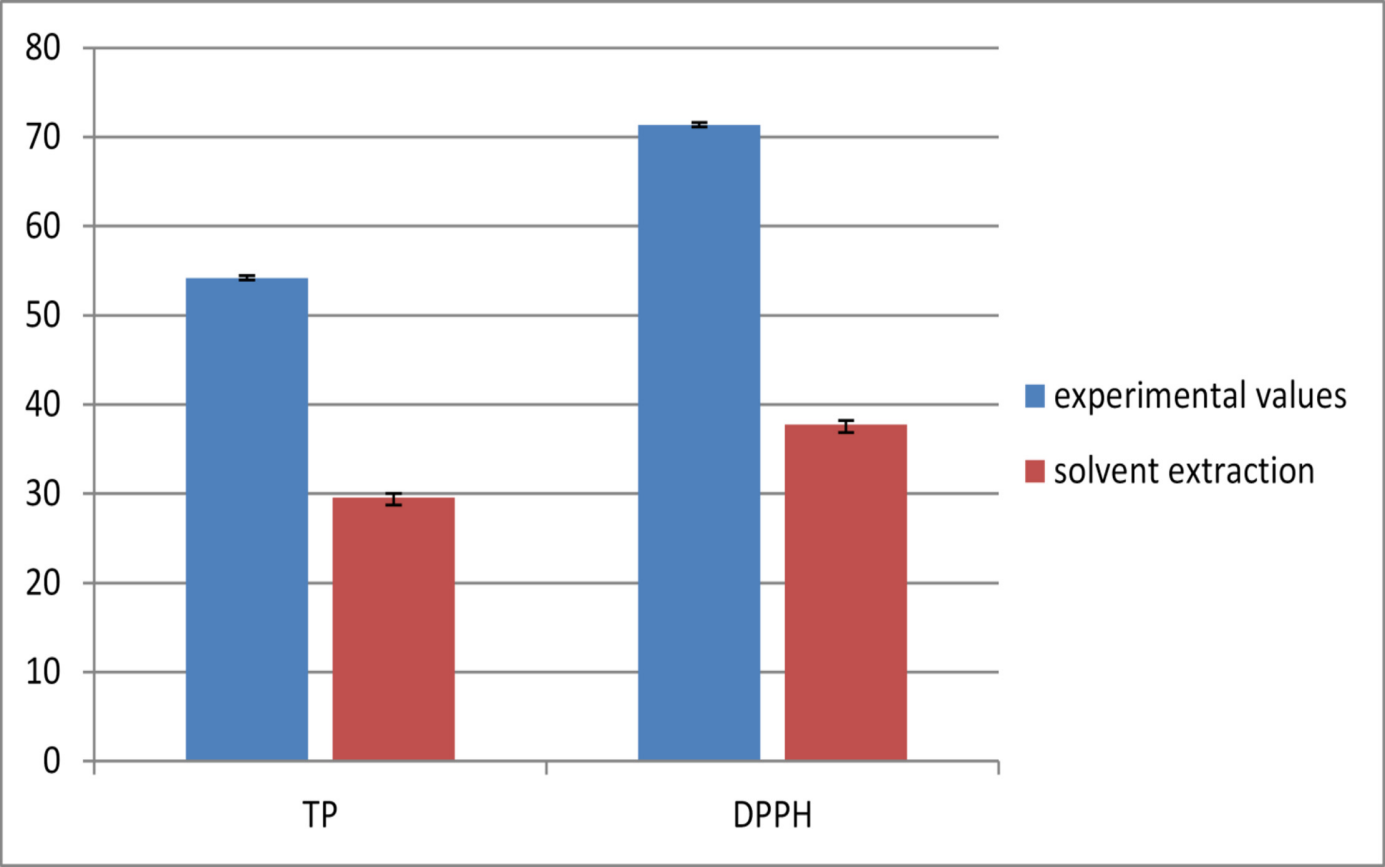
Experimental values and solvent extraction of TP(mg/100 g gallic acid) and DPPH (%) obtained for peach extracts.

In order to evaluate and confirm the validity of the models, a comparison of the predicted values and the experimental results was assigned. Hence, two optimal extraction parameters were established for peach experiments: (1) For total phenolics; modify the extraction temperature of 41.53 to 42°C, and the extraction power from 43.99% to 44%; and extraction time from 27.86 min to 28 min. (2) For rate of DPPH reduction; modify the extraction temperature of 41.60 C to 42°C and modify the extraction power of 44.88% to 45%; and the extraction time from 27.49min to 27 min. The results are shown in Fig 8 with the amounts of total phenolics and % DPPH under the optimal conditions and solvent extraction conditions. The results showed that there was no significant difference (p > 0.05) between the experimental and predicted values of total phenolics while the rate of DPPH was statistically significant (p < 0.05) between the experimental and predicted values.

### Morphological analysis

**Fig 9:**
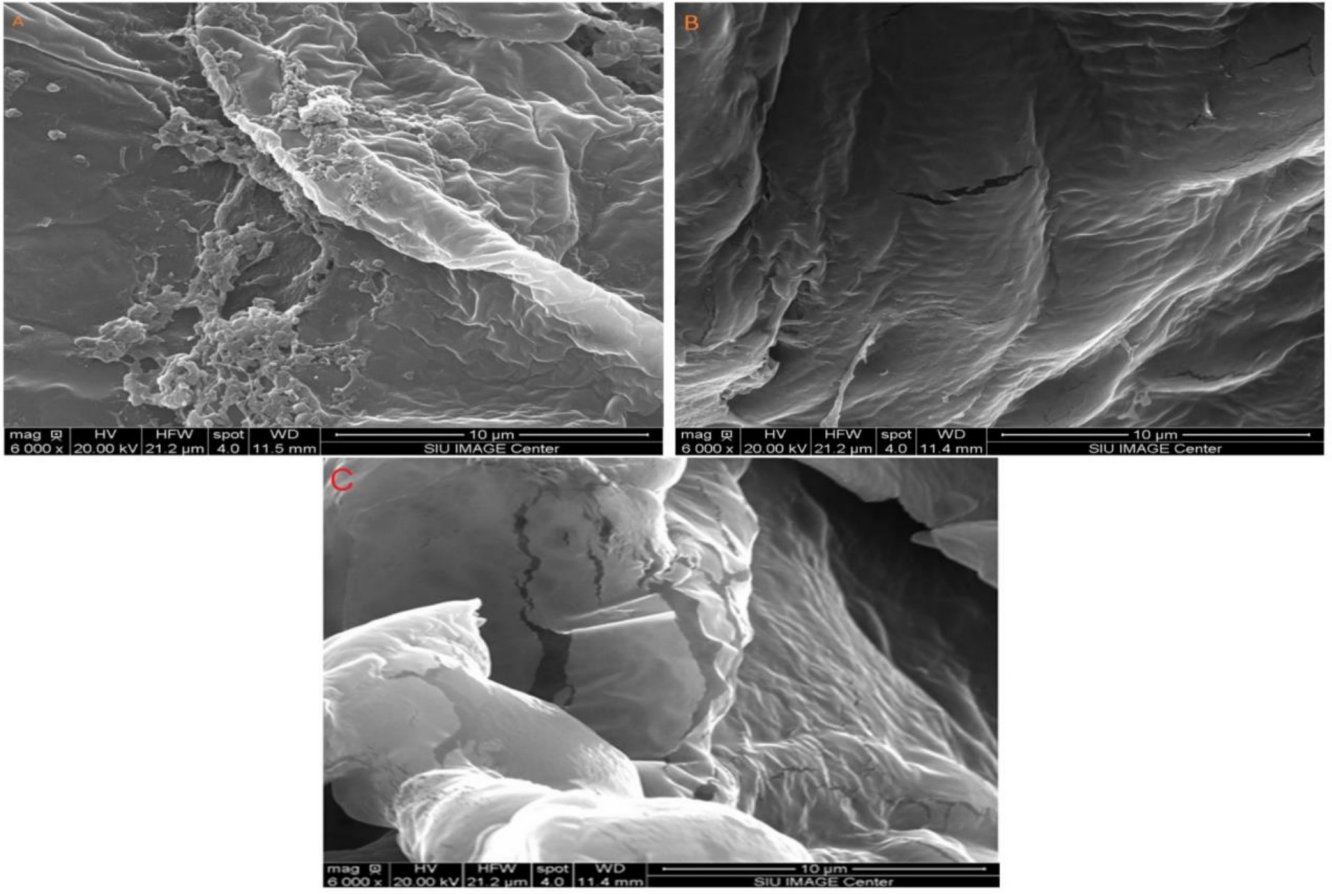
SEM images of pumpkin samples, Panel (A) a non-treated sample. Panel (B) a sample after solvent extraction. Panel (C) a sample after ultrasonic treatment.

The SEM images for pumpkin, and peach samples were shown before and after processing. The images of pumpkin (Fig 7) illustrate the structural changes in the samples. The non-treated material and the solvent extraction processed material are shown in Fig 9A and 9B, respectively. It appears that cell walls of pumpkin sample were not completely damaged while the ultrasonic treatment cells were highly damaged Fig 9C.

**Fig 10:**
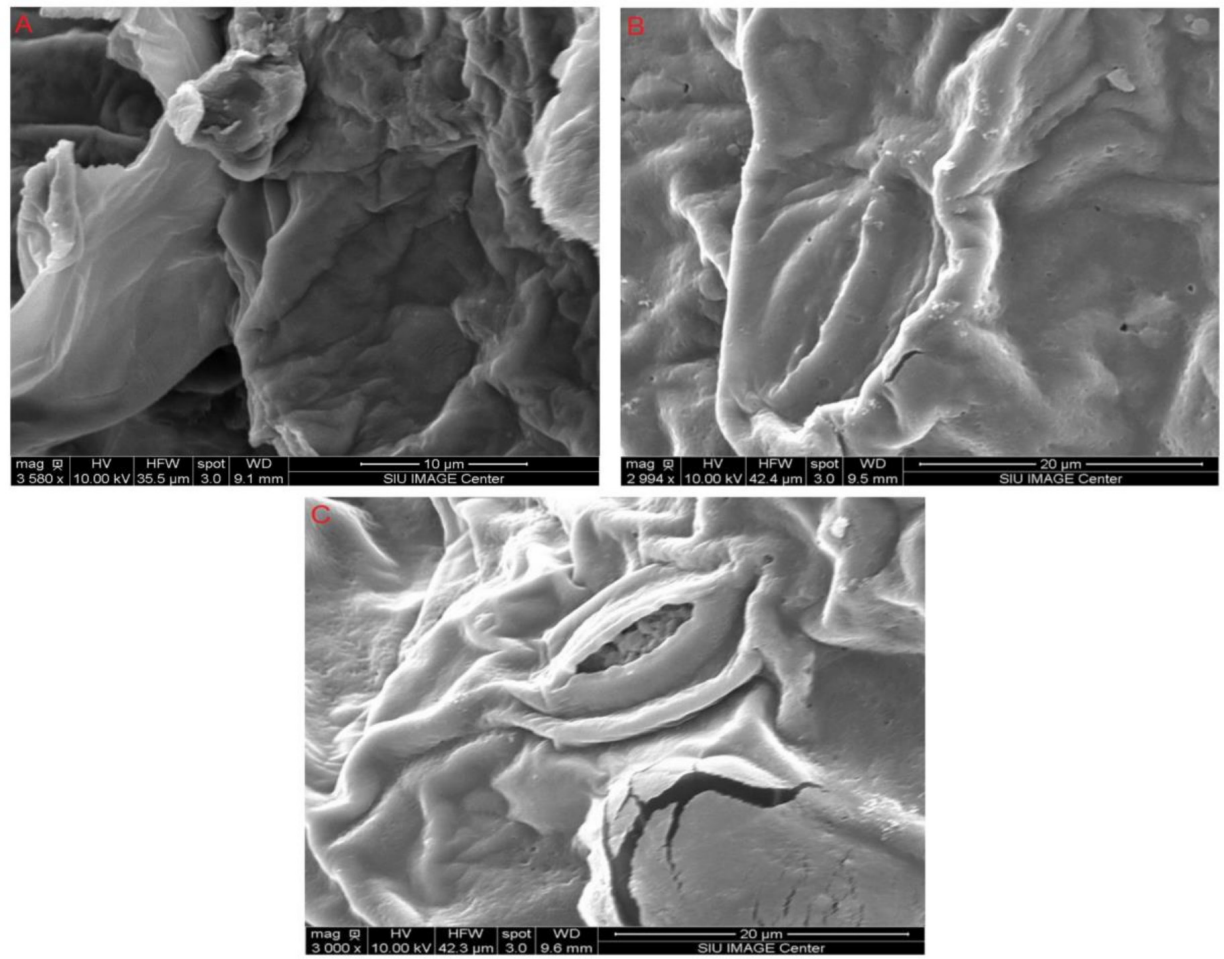
SEM images peach samples. Panel (A) a non-treated (B) a sample after solvent extraction. Panel (C) a sample after ultrasonic treatment sample.

Scanning electron micrographs of peaches samples are illustrated in Fig 10. There was no huge different between non-treated material (Fig 10A) and the solvent extraction processed material (Fig 10B). In contrast, ultrasonic treatment caused a lot of the cell damage (Fig 10C). Therefore, positive effects of ultrasonic processing on increasing cell damage, and increasing the rate of mass transfer for bioactive compounds were inferred.

### Studying the changes in chemical structure (FTIR)

**Fig 11:**
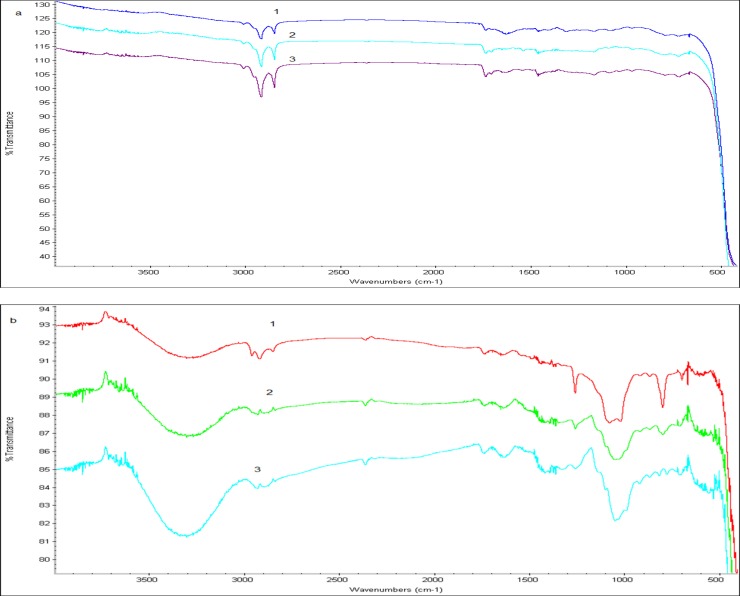
FTIR spectra. Panel (a) Samples of pumpkin. Panel (b) samples of peach. Line (1) was non-processed, (2) was solvent extracted and (3) was ultrasonically processed

The spectrums for pumpkin, and peach samples were shown before and after processing in order to catch if there will be any changes in the chemical structures. FTIR spectra are plotted in Fig 11A and 11B for pumpkin and peach, respectively. According to these results, there was no significantly change either by ultrasonic processing (line 3) or solvent extraction (line 2) compared to control samples (line 1) for pumpkin and peach Fig 11).

## Conclusions

Here, the ultrasound-assisted extraction was used to optimize the yield of phenolic compounds and the rate of free radical scavenging (% DPPH) from pumpkin and peach extracts. In both total phenolic content and the rate of free radical scavenging the regression models were significant and the lack-of-fits were insignificant. The optimal condition for the phenolic compounds and the rate of DPPH free radical scavenging from pumpkins extracts was found to be: at 41.45°C using extraction power of 44.60% and extraction time of 25.67 min; and extraction temperature of 40.99°C using extraction power of 56.01% and extraction time of 25.71 min respectively. However, the optimal conditions for peach extracts were obtained with an extraction temperature of 41.53°C, extraction power of 43.99% and extraction time of 27.86 min for phenolics. Free radical scavenging was optimal at extraction temperature of 41.60°C, extraction power of 44.88% and extraction time of 27.49 min respectively. The results showed that ultrasonic processing was powerful to cause damage in cells for all treated samples (pumpkin, peach) while the FTIR spectra did not show any significant change in chemical structural by either ultrasonic processing or solvent extraction method. It was concluded that UAE extraction was a significant improvement over conventional techniques. The improvements attributable to UAE were predicted in earlier works [17–20] and reviews [18, 38] but were realized in this study for two fruit crops of worldwide importance. We could not distinguish among the causes of the improvement in this study among increasing the extraction efficiency and increasing the bioactivity of the phenolics [38]. However, the FTIR spectra did not show any significant changes in chemical structures caused by either ultrasonic processing or solvent extraction. In future, we will examine the efficacy of quantified aliquots of these extracts in inhibiting the growth of common food borne pathogens to determine the biological optima for extractions.

## Supporting Information

S1 File Table A: Raw data of total phenol (TP) mg/ 100 g gallic acid and antioxidant activity (DPPH %) for peaches extracts. Table B: Raw data of TP mg/ 100 g gallic acid and antioxidant activity (DPPH %) for pumpkin extracts. Table C: Analysis of variance table for determination of DPPH for peach (partial sum of squares—type III). Table D: Analysis of variance table for determination of TP for peach (partial sum of squares—type III). Table E: Analysis of variance table for determination of DPPH for pumpkin (partial sum of squares—type III). Table F: Analysis of variance table for determination of TP for pumpkin (partial sum of squares—type III).
(DOCX)
