# Supplementary material for: Ultrasound Assisted Extraction of Phenolic Compounds from Peaches and Pumpkins
Source: PLoS One. 2016 Feb 17;11(2):e0148758. doi: 10.1371/journal.pone.0148758 (PMC4757553; doi:10.1371/journal.pone.0148758)
Supplement: S1 File — Table A: Raw data of total phenol (TP) mg/ 100 g gallic acid and antioxidant activity (DPPH %) for peaches extracts. Table B: Raw data of TP mg/ 100 g gallic acid and antioxidant activity (DPPH %) for pumpkin extracts. Table C: Analysis of variance table for determination of DPPH for peach (partial sum of squares—type III). Table D: Analysis of variance table for determination of TP for peach (partial sum of squares—type III). Table E: Analysis of variance table for determination of DPPH for pumpkin (partial sum of squares—type III). Table F: Analysis of variance table for determination of TP for pumpkin (partial sum of squares—type III). (DOCX) [file pone.0148758.s001.docx]

Supporting Information S1: Table A: Raw data of total phenol (TP) mg/ 100 g Gallic acid and antioxidant activity (DPPH %) for peaches extracts

|  | Factor 1 | Factor 2 | Factor 3 | Response 1 | Response 2 |
| --- | --- | --- | --- | --- | --- |
| Run | A:temperature | B:power | C:time | DPPH | total phenol |
|  | c | w | min |  |  |
| 1 | 40.00 | 30.00 | 20.00 | 72.09 | 54.11 |
| 2 | 50.00 | 30.00 | 20.00 | 70.12 | 52.14 |
| 3 | 40.00 | 70.00 | 20.00 | 71.17 | 53.19 |
| 4 | 50.00 | 70.00 | 20.00 | 70.11 | 51.13 |
| 5 | 40.00 | 70.00 | 30.00 | 71.07 | 52.09 |
| 6 | 40.00 | 50.00 | 10.00 | 71.11 | 52.13 |
| 7 | 50.00 | 30.00 | 20.00 | 71.17 | 52.18 |
| 8 | 30.00 | 70.00 | 10.00 | 69.09 | 50.11 |
| 9 | 30.00 | 50.00 | 30.00 | 69.25 | 51.27 |
| 10 | 50.00 | 50.00 | 20.00 | 71.13 | 52.15 |
| 11 | 50.00 | 70.00 | 10.00 | 68.97 | 49.88 |
| 12 | 50.00 | 30.00 | 10.00 | 70.07 | 51.11 |
| 13 | 40.00 | 30.00 | 30.00 | 72.99 | 53.88 |
| 14 | 40.00 | 30.00 | 30.00 | 72.65 | 53.53 |
| 15 | 30.00 | 50.00 | 30.00 | 70.22 | 51.33 |
| 16 | 50.00 | 30.00 | 30.00 | 72.28 | 53.42 |
| 17 | 30.00 | 30.00 | 20.00 | 70.25 | 51.23 |
| 18 | 40.00 | 30.00 | 30.00 | 72.44 | 53.46 |
| 19 | 50.00 | 30.00 | 10.00 | 70.24 | 51.26 |
| 20 | 50.00 | 70.00 | 10.00 | 69.33 | 50.35 |
| 21 | 30.00 | 70.00 | 20.00 | 71.03 | 52.07 |
| 22 | 40.00 | 50.00 | 10.00 | 73.23 | 54.31 |
| 23 | 40.00 | 70.00 | 30.00 | 72.31 | 54.13 |
| 24 | 40.00 | 70.00 | 10.00 | 72.22 | 53.76 |
| 25 | 30.00 | 50.00 | 20.00 | 70.89 | 51.77 |
| 26 | 40.00 | 50.00 | 10.00 | 70.33 | 51.66 |
| 27 | 30.00 | 70.00 | 20.00 | 70.22 | 52.31 |
| 28 | 40.00 | 70.00 | 20.00 | 72.31 | 52.29 |
| 29 | 40.00 | 30.00 | 20.00 | 70.23 | 51.26 |
| 30 | 40.00 | 70.00 | 20.00 | 71.65 | 54.55 |
| 31 | 50.00 | 30.00 | 30.00 | 72.33 | 53.43 |
| 32 | 40.00 | 50.00 | 20.00 | 71.88 | 52.78 |
| 33 | 50.00 | 50.00 | 20.00 | 71.22 | 52.12 |
| 34 | 30.00 | 30.00 | 30.00 | 69.94 | 49.84 |
| 35 | 50.00 | 30.00 | 20.00 | 72.65 | 53.75 |
| 36 | 50.00 | 50.00 | 10.00 | 70.96 | 51.86 |
| 37 | 40.00 | 30.00 | 10.00 | 72 | 53.39 |
| 38 | 40.00 | 70.00 | 10.00 | 71.29 | 53.42 |
| 39 | 50.00 | 50.00 | 10.00 | 70.24 | 51.31 |
| 40 | 50.00 | 70.00 | 20.00 | 70.28 | 51.27 |
| 41 | 30.00 | 30.00 | 30.00 | 68.55 | 51.75 |
| 42 | 50.00 | 30.00 | 30.00 | 71.99 | 52.86 |
| 43 | 50.00 | 50.00 | 30.00 | 71.88 | 52.79 |
| 44 | 40.00 | 30.00 | 20.00 | 71.99 | 52.92 |
| 45 | 40.00 | 70.00 | 10.00 | 72.39 | 53.4 |
| 46 | 30.00 | 50.00 | 20.00 | 70.95 | 51.77 |
| 47 | 40.00 | 50.00 | 30.00 | 73.14 | 55.11 |
| 48 | 30.00 | 70.00 | 10.00 | 69 | 51.36 |
| 49 | 50.00 | 50.00 | 10.00 | 71.01 | 52.15 |
| 50 | 30.00 | 70.00 | 20.00 | 70.19 | 51.28 |
| 51 | 30.00 | 70.00 | 30.00 | 71.08 | 52.19 |
| 52 | 40.00 | 30.00 | 10.00 | 72.15 | 53.23 |
| 53 | 40.00 | 50.00 | 30.00 | 75.2 | 55.2 |
| 54 | 40.00 | 30.00 | 10.00 | 72.51 | 53.51 |
| 55 | 30.00 | 30.00 | 20.00 | 70.21 | 49.19 |
| 56 | 50.00 | 50.00 | 30.00 | 72.9 | 53.82 |
| 57 | 30.00 | 30.00 | 20.00 | 69.34 | 50.29 |
| 58 | 40.00 | 70.00 | 30.00 | 71.9 | 52.77 |
| 59 | 30.00 | 30.00 | 10.00 | 69.03 | 50.13 |
| 60 | 40.00 | 50.00 | 20.00 | 73.81 | 55.68 |
| 61 | 30.00 | 30.00 | 10.00 | 69.15 | 50.09 |
| 62 | 40.00 | 50.00 | 30.00 | 73.04 | 54.15 |
| 63 | 30.00 | 50.00 | 10.00 | 67.73 | 51.08 |
| 64 | 30.00 | 70.00 | 10.00 | 69.24 | 50.44 |
| 65 | 30.00 | 70.00 | 30.00 | 69.46 | 50.51 |
| 66 | 50.00 | 70.00 | 10.00 | 69.89 | 50.72 |
| 67 | 50.00 | 70.00 | 30.00 | 69.58 | 50.97 |
| 68 | 30.00 | 30.00 | 10.00 | 69.4 | 50.46 |
| 69 | 50.00 | 70.00 | 30.00 | 69.68 | 50.77 |
| 70 | 50.00 | 70.00 | 20.00 | 70.28 | 51.38 |
| 71 | 50.00 | 50.00 | 30.00 | 71.29 | 50.21 |
| 72 | 30.00 | 50.00 | 10.00 | 69.71 | 49.53 |
| 73 | 30.00 | 50.00 | 10.00 | 69.99 | 49.62 |
| 74 | 50.00 | 50.00 | 20.00 | 71.88 | 52.71 |
| 75 | 30.00 | 50.00 | 30.00 | 70.88 | 51.83 |
| 76 | 50.00 | 70.00 | 30.00 | 70.79 | 51.93 |
| 77 | 30.00 | 30.00 | 30.00 | 69.85 | 50.76 |
| 78 | 50.00 | 30.00 | 10.00 | 69.78 | 50.55 |
| 79 | 30.00 | 50.00 | 20.00 | 71.01 | 51.66 |
| 80 | 40.00 | 50.00 | 20.00 | 75.76 | 54.79 |
| 81 | 30.00 | 70.00 | 30.00 | 70.33 | 51.36 |

Supporting Information S1: Table B: Raw data of total phenol (TP) mg/ 100 g Gallic acid and antioxidant activity (DPPH %) for pumpkin extracts

|  | Factor 1 | Factor 2 | Factor 3 | Response 1 | Response 2 |
| --- | --- | --- | --- | --- | --- |
| Run | A:temperature | B:power | C:time | TP | DPPH |
|  | C | % | min | % |  |
| 1 | 40.00 | 30.00 | 20.00 | 43.09 | 63.09 |
| 2 | 50.00 | 30.00 | 20.00 | 41.12 | 61.12 |
| 3 | 40.00 | 70.00 | 20.00 | 42.17 | 64.23 |
| 4 | 50.00 | 70.00 | 20.00 | 40.11 | 60.17 |
| 5 | 40.00 | 70.00 | 30.00 | 41.07 | 63.11 |
| 6 | 40.00 | 50.00 | 10.00 | 41.11 | 65.11 |
| 7 | 50.00 | 30.00 | 20.00 | 41.17 | 61.17 |
| 8 | 30.00 | 70.00 | 10.00 | 39.09 | 60.09 |
| 9 | 30.00 | 50.00 | 30.00 | 40.25 | 60.15 |
| 10 | 50.00 | 50.00 | 20.00 | 41.13 | 61.13 |
| 11 | 50.00 | 70.00 | 10.00 | 38.97 | 58.97 |
| 12 | 50.00 | 30.00 | 10.00 | 40.07 | 60.07 |
| 13 | 40.00 | 30.00 | 30.00 | 42.99 | 62.99 |
| 14 | 40.00 | 30.00 | 30.00 | 42.65 | 62.65 |
| 15 | 30.00 | 50.00 | 30.00 | 40.22 | 60.12 |
| 16 | 50.00 | 30.00 | 30.00 | 42.28 | 62.28 |
| 17 | 30.00 | 30.00 | 20.00 | 40.25 | 60.15 |
| 18 | 40.00 | 30.00 | 30.00 | 42.44 | 62.44 |
| 19 | 50.00 | 30.00 | 10.00 | 40.24 | 60.17 |
| 20 | 50.00 | 70.00 | 10.00 | 39.33 | 60.11 |
| 21 | 30.00 | 70.00 | 20.00 | 41.03 | 61.15 |
| 22 | 40.00 | 50.00 | 10.00 | 43.23 | 61.09 |
| 23 | 40.00 | 70.00 | 30.00 | 43.31 | 63.17 |
| 24 | 40.00 | 70.00 | 10.00 | 42.22 | 62.19 |
| 25 | 30.00 | 50.00 | 20.00 | 40.89 | 60.98 |
| 26 | 40.00 | 50.00 | 10.00 | 40.33 | 61.28 |
| 27 | 30.00 | 70.00 | 20.00 | 41.22 | 61.19 |
| 28 | 40.00 | 70.00 | 20.00 | 42.31 | 64.26 |
| 29 | 40.00 | 30.00 | 20.00 | 40.23 | 60.11 |
| 30 | 40.00 | 70.00 | 20.00 | 43.65 | 63.55 |
| 31 | 50.00 | 30.00 | 30.00 | 42.33 | 62.2 |
| 32 | 40.00 | 50.00 | 20.00 | 41.88 | 64.8 |
| 33 | 50.00 | 50.00 | 20.00 | 41.22 | 61.2 |
| 34 | 30.00 | 30.00 | 30.00 | 38.94 | 57.94 |
| 35 | 50.00 | 30.00 | 20.00 | 42.65 | 62.65 |
| 36 | 50.00 | 50.00 | 10.00 | 40.96 | 59.96 |
| 37 | 40.00 | 30.00 | 10.00 | 42.29 | 62.09 |
| 38 | 40.00 | 70.00 | 10.00 | 42.37 | 62.24 |
| 39 | 50.00 | 50.00 | 10.00 | 40.24 | 60.14 |
| 40 | 50.00 | 70.00 | 20.00 | 40.28 | 60.24 |
| 41 | 30.00 | 30.00 | 30.00 | 40.88 | 59.99 |
| 42 | 50.00 | 30.00 | 30.00 | 41.98 | 62.19 |
| 43 | 50.00 | 50.00 | 30.00 | 41.88 | 61.8 |
| 44 | 40.00 | 30.00 | 20.00 | 41.99 | 62.09 |
| 45 | 40.00 | 70.00 | 10.00 | 42.39 | 62.29 |
| 46 | 30.00 | 50.00 | 20.00 | 40.95 | 59.97 |
| 47 | 40.00 | 50.00 | 30.00 | 44.05 | 62.11 |
| 48 | 30.00 | 70.00 | 10.00 | 40.22 | 60.15 |
| 49 | 50.00 | 50.00 | 10.00 | 41.01 | 60.19 |
| 50 | 30.00 | 70.00 | 20.00 | 40.19 | 60.19 |
| 51 | 30.00 | 70.00 | 30.00 | 41.08 | 61.08 |
| 52 | 40.00 | 30.00 | 10.00 | 42.15 | 62.15 |
| 53 | 40.00 | 50.00 | 30.00 | 45.2 | 65.2 |
| 54 | 40.00 | 30.00 | 10.00 | 42.51 | 62.51 |
| 55 | 30.00 | 30.00 | 20.00 | 40.19 | 60.19 |
| 56 | 50.00 | 50.00 | 30.00 | 42.88 | 61.96 |
| 57 | 30.00 | 30.00 | 20.00 | 39.31 | 60.22 |
| 58 | 40.00 | 70.00 | 30.00 | 41.88 | 62.95 |
| 59 | 30.00 | 30.00 | 10.00 | 39.01 | 57.96 |
| 60 | 40.00 | 50.00 | 20.00 | 44.79 | 64.88 |
| 61 | 30.00 | 30.00 | 10.00 | 39.13 | 57.11 |
| 62 | 40.00 | 50.00 | 30.00 | 43.02 | 65.98 |
| 63 | 30.00 | 50.00 | 10.00 | 37.71 | 57.84 |
| 64 | 30.00 | 70.00 | 10.00 | 39.22 | 60.19 |
| 65 | 30.00 | 70.00 | 30.00 | 39.44 | 60.33 |
| 66 | 50.00 | 70.00 | 10.00 | 38.88 | 57.92 |
| 67 | 50.00 | 70.00 | 30.00 | 39.56 | 61.02 |
| 68 | 30.00 | 30.00 | 10.00 | 38.38 | 57.95 |
| 69 | 50.00 | 70.00 | 30.00 | 39.66 | 60.95 |
| 70 | 50.00 | 70.00 | 20.00 | 40.26 | 61.26 |
| 71 | 50.00 | 50.00 | 30.00 | 41.28 | 62.08 |
| 72 | 30.00 | 50.00 | 10.00 | 38.69 | 57.89 |
| 73 | 30.00 | 50.00 | 10.00 | 38.99 | 57.99 |
| 74 | 50.00 | 50.00 | 20.00 | 41.99 | 61.99 |
| 75 | 30.00 | 50.00 | 30.00 | 40.31 | 60.21 |
| 76 | 50.00 | 70.00 | 30.00 | 40.77 | 62.77 |
| 77 | 30.00 | 30.00 | 30.00 | 39.88 | 58.88 |
| 78 | 50.00 | 30.00 | 10.00 | 39.85 | 65.85 |
| 79 | 30.00 | 50.00 | 20.00 | 40.99 | 59.99 |
| 80 | 40.00 | 50.00 | 20.00 | 44.88 | 64.88 |
| 81 | 30.00 | 70.00 | 30.00 | 40.23 | 61.13 |

| Supporting Information S1: Table C: Analysis of variance table for determination of DPPH for peach [Partial sum of squares - Type III] | | | | | | |
| --- | --- | --- | --- | --- | --- | --- |
|  | **Sum of** |  | **Mean** | **F** | **p-value** |  |
| **Source** | **Squares** | **df** | **Square** | **Value** | **Prob > F** |  |
| Model | 120.58 | 9 | 13.40 | 18.45 | < 0.0001 | significant |
| *A-temperature* | *12.58* | *1* | *12.58* | *17.32* | *< 0.0001* |  |
| *B-power* | *2.06* | *1* | *2.06* | *2.83* | *0.0967* |  |
| *C-time* | *13.46* | *1* | *13.46* | *18.53* | *< 0.0001* |  |
| *AB* | *6.79* | *1* | *6.79* | *9.36* | *0.0031* |  |
| *AC* | *0.70* | *1* | *0.70* | *0.96* | *0.3305* |  |
| *BC* | *0.42* | *1* | *0.42* | *0.58* | *0.4470* |  |
| *A^2* | *71.57* | *1* | *71.57* | *98.56* | *< 0.0001* |  |
| *B^2* | *10.39* | *1* | *10.39* | *14.30* | *0.0003* |  |
| *C^2* | *2.61* | *1* | *2.61* | *3.59* | *0.0621* |  |
| Residual | 51.56 | 71 | 0.73 |  |  |  |
| *Lack of Fit* | *17.13* | *17* | *1.01* | *1.58* | *0.1026* | *not significant* |
| *Pure Error* | *34.43* | *54* | *0.64* |  |  |  |
| Cor Total | 172.14 | 80 |  |  |  |  |

| Supporting Information S1: Table D: Analysis of variance table for determination of TP for peach [Partial sum of squares - Type III] | | | | | | |
| --- | --- | --- | --- | --- | --- | --- |
|  | **Sum of** |  | **Mean** | **F** | **p-value** |  |
| **Source** | **Squares** | **df** | **Square** | **Value** | **Prob > F** |  |
| Model | 122.98 | 9 | 13.66 | 18.77 | < 0.0001 | significant |
| *A-temperature* | *9.79* | *1* | *9.79* | *13.44* | *0.0005* |  |
| *B-power* | *0.31* | *1* | *0.31* | *0.43* | *0.5153* |  |
| *C-time* | *11.15* | *1* | *11.15* | *15.32* | *0.0002* |  |
| *AB* | *11.32* | *1* | *11.32* | *15.55* | *0.0002* |  |
| *AC* | *0.25* | *1* | *0.25* | *0.34* | *0.5610* |  |
| *BC* | *0.97* | *1* | *0.97* | *1.34* | *0.2514* |  |
| *A^2* | *82.99* | *1* | *82.99* | *113.99* | *< 0.0001* |  |
| *B^2* | *4.24* | *1* | *4.24* | *5.83* | *0.0183* |  |
| *C^2* | *1.95* | *1* | *1.95* | *2.67* | *0.1064* |  |
| Residual | 51.69 | 71 | 0.73 |  |  |  |
| *Lack of Fit* | *14.16* | *17* | *0.83* | *1.20* | *0.2970* | *not significant* |
| *Pure Error* | *37.53* | *54* | *0.69* |  |  |  |
| Cor Total | 174.67 | 80 |  |  |  |  |

| Supporting Information S1: Table E: Analysis of variance table for determination of DPPH for pumpkin [Partial sum of squares - Type III] |
| --- |
|  |

|  | | | | | | |
| --- | --- | --- | --- | --- | --- | --- |
|  | **Sum of** |  | **Mean** | **F** | **p-value** |  |
| **Source** | **Squares** | **df** | **Square** | **Value** | **Prob > F** |  |
| Model | 218.94 | 9 | 24.33 | 19.66 | < 0.0001 | significant |
| *A-temperature* | *30.42* | *1* | *30.42* | *24.58* | *< 0.0001* |  |
| *B-power* | *1.40* | *1* | *1.40* | *1.13* | *0.2914* |  |
| *C-time* | *24.24* | *1* | *24.24* | *19.59* | *< 0.0001* |  |
| *AB* | *24.01* | *1* | *24.01* | *19.40* | *< 0.0001* |  |
| *AC* | *0.041* | *1* | *0.041* | *0.033* | *0.8567* |  |
| *BC* | *1.23* | *1* | *1.23* | *1.00* | *0.3218* |  |
| *A^2* | *128.52* | *1* | *128.52* | *103.85* | *< 0.0001* |  |
| *B^2* | *1.73* | *1* | *1.73* | *1.40* | *0.2413* |  |
| *C^2* | *7.36* | *1* | *7.36* | *5.94* | *0.0173* |  |
| Residual | 87.87 | 71 | 1.24 |  |  |  |
| *Lack of Fit* | *30.47* | *17* | *1.79* | *1.69* | *0.0747* | *not significant* |
| *Pure Error* | *57.40* | *54* | *1.06* |  |  |  |
| Cor Total | 306.81 | 80 |  |  |  |  |

| Supporting Information S1: Table F: Analysis of variance table for determination of TP for pumpkin [Partial sum of squares - Type III] |
| --- |
| \|  \| \| \| \| \| \| \| \| --- \| --- \| --- \| --- \| --- \| --- \| --- \| \|  \| **Sum of** \|  \| **Mean** \| **F** \| **p-value** \|  \| \| **Source** \| **Squares** \| **df** \| **Square** \| **Value** \| **Prob > F** \|  \| \| Model \| 146.87 \| 9 \| 16.32 \| 22.94 \| < 0.0001 \| significant \| \| *A-temperature* \| *11.96* \| *1* \| *11.96* \| *16.81* \| *0.0001* \|  \| \| *B-power* \| *0.93* \| *1* \| *0.93* \| *1.31* \| *0.2565* \|  \| \| *C-time* \| *18.81* \| *1* \| *18.81* \| *26.44* \| *< 0.0001* \|  \| \| *AB* \| *10.69* \| *1* \| *10.69* \| *15.03* \| *0.0002* \|  \| \| *AC* \| *0.14* \| *1* \| *0.14* \| *0.20* \| *0.6537* \|  \| \| *BC* \| *1.15* \| *1* \| *1.15* \| *1.61* \| *0.2080* \|  \| \| *A^2* \| *91.29* \| *1* \| *91.29* \| *128.33* \| *< 0.0001* \|  \| \| *B^2* \| *6.03* \| *1* \| *6.03* \| *8.47* \| *0.0048* \|  \| \| *C^2* \| *5.87* \| *1* \| *5.87* \| *8.25* \| *0.0054* \|  \| \| Residual \| 50.51 \| 71 \| 0.71 \|  \|  \|  \| \| *Lack of Fit* \| *18.30* \| *17* \| *1.08* \| *1.80* \| *0.0518* \| *not significant* \| \| *Pure Error* \| *32.21* \| *54* \| *0.60* \|  \|  \|  \| \| Cor Total \| 197.37 \| 80 \|  \|  \|  \|  \| |
